# Quantifying the Surface Strain Field Induced by Active Sources with Distributed Acoustic Sensing: Theory and Practice

**DOI:** 10.3390/s22124589

**Published:** 2022-06-17

**Authors:** Peter G. Hubbard, Joseph P. Vantassel, Brady R. Cox, James W. Rector, Michael B. S. Yust, Kenichi Soga

**Affiliations:** 1 Department of Civil and Environmental Engineering, University of California, Berkeley, CA 94720, USA; jwrector@berkeley.edu (J.W.R.); soga@berkeley.edu (K.S.); 2 Department of Civil, Architectural, and Environmental Engineering, The University of Texas at Austin, Austin, TX 78712, USA; jvantassel@utexas.edu (J.P.V.); yustm@utexas.edu (M.B.S.Y.); 3 Department of Civil and Environmental Engineering, Utah State University, Logan, UT 84322, USA; brady.cox@usu.edu

**Keywords:** DAS, geophones, transfer function, wave propagation, strain measurement, DFOS

## Abstract

Quantitative dynamic strain measurements of the ground would be useful for engineering scale problems such as monitoring for natural hazards, soil-structure interaction studies, and non-invasive site investigation using full waveform inversion (FWI). Distributed acoustic sensing (DAS), a promising technology for these purposes, needs to be better understood in terms of its directional sensitivity, spatial position, and amplitude for application to engineering-scale problems. This study investigates whether the physical measurements made using DAS are consistent with the theoretical transfer function, reception patterns, and experimental measurements of ground strain made by geophones. Results show that DAS and geophone measurements are consistent in both phase and amplitude for broadband (10 s of Hz), high amplitude (10 s of microstrain), and complex wavefields originating from different positions around the array when: (1) the DAS channels and geophone locations are properly aligned, (2) the DAS cable provides good deformation coupling to the internal optical fiber, (3) the cable is coupled to the ground through direct burial and compaction, and (4) laser frequency drift is mitigated in the DAS measurements. The transfer function of DAS arrays is presented considering the gauge length, pulse shape, and cable design. The theoretical relationship between DAS-measured and pointwise strain for vertical and horizontal active sources is introduced using 3D elastic finite-difference simulations. The implications of using DAS strain measurements are discussed including directionality and magnitude differences between the actual and DAS-measured strain fields. Estimating measurement quality based on the wavelength-to-gauge length ratio for field data is demonstrated. A method for spatially aligning the DAS channels with the geophone locations at tolerances less than the spatial resolution of a DAS system is proposed.

## 1. Introduction

Distributed acoustic sensing (DAS) is a technique that dynamically measures the change in length of sections of optical fiber. This is done by examining the optical path difference (OPD), or the change in distance that light travels, of Rayleigh backscatter over a length of optical fiber [1]. Rayleigh backscatter is light that propagates in the opposite direction of source laser light at the same frequency. It is caused primarily by the incident light interacting with random inhomogeneities contained within the fiber [2]. Rayleigh backscatter that has originated from different locations along an optical fiber is combined to create an interference (i.e., a superposition of wave energy). The interference pattern is used to determine the Rayleigh backscatter’s phase difference between the two backscatter sources, and in turn the evolution of the OPD between them. The average straight-line distance between the origins of the Rayleigh backscatter is called the gauge length (*g*). In the case of DAS, the light phase difference is the measurement output by the instrument, expressed as radians relative to the source light’s average wavelength. This is converted to OPD through transformations discussed later. The phase measurements are proportional to changes in length of the fiber (due to thermal and mechanical strain), fiber refractive index, and incident light wavelength [3]. Since the measured OPD is relative to the light’s wavelength, the corresponding measurements of length change (ΔL), or axial/normal strain (ε=ΔL/g) if divided by the gauge length, are extremely small. Strain measurements are examined dynamically and can be made as sensitive as $0.6p\varepsilon /\sqrt{\mathrm{Hz}}$ [4].

Sources of DAS strain measurement error that are of large concern to an end user are laser frequency drift, temperature-induced refractive index changes, and phase determination errors [5,6,7]. Other noise sources exist, including thermal effects on the photodetector, shot, and amplifier noise [8], though these sources are typically not addressed by a user due to limited knowledge of the optoelectronics of DAS systems. These latter noise sources may be considered by DAS manufacturers when determining optimal operating temperature, maximum measurement distance, and amplifier configurations for a system [9,10]. Laser frequency drift, temperature-induced refractive index changes and phase errors, on the other hand, are more pertinent to users because of their obvious effects on the amplitude of DC-coupled measurements output by a commercial DAS instrument.

Laser frequency drift is the slow variation of the wavelength of light interrogating a sensing fiber. Any deviation from the average wavelength at the source will manifest in the phase measurements as a change. Similarly, temperature-induced refractive index changes caused by environmental fluctuations as small as a millikelvin result in changes to the OPD at low frequencies. These types of strain measurement error can be reduced by high pass filtering or time-differentiating the phase data, which attenuates signals near DC [1].

Phase determination errors can be the result of incorrect unwrapping, or sometimes called demodulation [11], of the circular phase. This can be due to phase changes that are too large between time samples to accurately track. It can also be the result of signal fading which causes low SNR and therefore a poorly determined phase measurement [12]. These phase errors typically manifest as jumps (step functions) in the time domain phase data, or broadband noise in the frequency domain. In this study, we demonstrate that: (i) the noise sources of laser frequency drift and temperature-induced refractive index changes can be mitigated by high pass filtering the data within a dynamic range of interest, and (ii) phase determination errors have a limited impact on the recorded waveforms if the maximum sampling rate (100 kHz) of the interrogator unit is employed (Optasense Inc.’s ODH-4 in the present study).

DAS has been used in the geophysical community for over a decade for measuring stress wave propagation. Specific use cases include DAS deployed in boreholes for conducting vertical seismic profiling (VSP) [13,14], within telecommunications conduit for ambient noise interferometry [15,16], and offshore for passive seismic monitoring [17,18]. DAS is widely accepted as a technique for detecting and measuring the phase behavior of body and surface waves. It has been described that the measurements made by DAS are approximately equal to average strain or average strain-rate [19], as defined by:

(1) $${\hat{\dot{\varepsilon }}}_{DAS}\approx \;\frac{\left[u\left(x+\frac{g}{2},t+dt\right)-u\left(x-\frac{g}{2},t+dt\right)\right]-\left[u\left(x+\frac{g}{2},t\right)-u\left(x-\frac{g}{2},t\right)\right]}{gdt}$$

where ${\hat{\dot{\varepsilon }}}_{DAS}$ is a DAS strain-rate measurement, *u* is the displacement in the direction of the cable (i.e., the *x*-direction), and dt is the time step between samples. It should be noted that Equation (1) assumes perfectly discrete scattering locations, when it is actually a distribution over the light pulse’s shape [20], which will be discussed more later. This has allowed for the approximate conversion of DAS-measured strain to pointwise particle velocity assuming non-dispersive signals and large wavelengths relative to the gauge length of the DAS system. This relationship is:

(2) $${\hat{\varepsilon }}_{DAS}\approx \frac{du}{dx}=\frac{du}{cdt}=\frac{v}{c}$$

where ${\hat{\varepsilon }}_{DAS}$ is a DAS strain measurement, *c* is the phase velocity of the propagating seismic wave and *v* is the particle velocity in the direction of the DAS measurement.

The relationship given in Equation (2) only works when the average strain is close to the pointwise strain ($\frac{du}{dx}$) and when *c* can be easily estimated for all frequency components within the measured signal. It is demonstrated effectively for isolated body wave arrivals within boreholes in Daley et al. [19]. Wang et al. [21] applied the relationships in both Equations (1) and (2) to a surface DAS array and co-located geophones to measure long-wavelength signals at the crustal scale. It was shown that the waveforms matched well for non-dispersive signals within a frequency band of 1–5 Hz and amplitudes less than 100 nε/s. *c* was estimated by examining the moveout of a constant phase across the array. This finding was significant for seismological applications of DAS, as the recorded signals were generated from a M_L_ 4.3 earthquake and recorded across a wide aperture array of both geophones and DAS. It was noted that the amplitudes for the first few cycles were approximately the same between DAS and geophones but began to deviate later in the waveform following the S-wave arrival. This was attributed to interference between P- and S-wave coda, and a misalignment of the geophone and DAS measurement locations. In this study, we will show that DAS measurements can be consistent with geophone measurements at an engineering scale regardless of wavefield complexities for broadband and high amplitude signals.

Most reported DAS applications have not examined the amplitude of the signals or put emphasis on converting them to physical units because the phase behavior of the measurements was of primary interest. For example, Mateeva et al. [22] presents one of the first explanations of DAS for seismic monitoring in boreholes, where a comparison between DAS and geophones is performed for picking body wave arrivals. This required identifying seismic phases in the raw data; the signal’s specific amplitude or units was not used. Song et al. [23] shows the use of DAS for measuring surface wave dispersion to invert the velocity structure of the near subsurface. A step in this process is normalizing the amplitude of the traces, which is a common step in surface wave processing. Vantassel et al. [24] demonstrated that measurements of surface wave dispersion using DAS and geophone data could be compared in their respective raw units and produce essentially equivalent results, if frequency-dependent normalization was applied to the dispersion images following processing. Since the phase component of the DAS measurement alone has served the needs of the geophysical community, there has been little effort to study and quantify the signal amplitude. However, for more advanced imaging techniques, such as full-waveform inversion (FWI), both phase and amplitude are required [25]. Geophysical research has begun to move in the direction of using DAS for FWI. For example, Egorov et al. [26] used DAS VSP data for FWI. The DAS data were converted to equivalent units as the geophones using Equation (2) and then a shaping filter was applied to better match the DAS and geophone traces. Eaid et al. [27] also conducted FWI using DAS in units of strain, but the FWI residuals were normalized by the $L_{2}$-norm of the DAS data, effectively removing its amplitude. Furthermore, to use DAS as an engineering tool to monitor soil–structure interaction and ground deformation the amplitude of the strain measurements must be verified and not subjectively shaped.

This study aims to provide a procedure for using DAS data as quantitative ground strain measurements for stress-wave-based imaging techniques, such as FWI, that do not rely on the subjective shaping of the signal’s amplitude, or for engineering problems like soil–structure interaction and ground deformation monitoring. Specifically, this study will first demonstrate how DAS data can be modeled as a finite-difference of the displacement field, and then how to practically implement a quantitative acquisition in the field. The implications of measuring a spatially differenced displacement field are explored through the transfer function of DAS and 3D finite difference simulations of seismic waves produced by active sources. These theoretical observations are compared with field-measured wavefields when an optical fiber is carefully coupled with the ground and seismic waves are created with active sources. The amplitudes of the strain measurements made with DAS are validated through quantitative comparison to geophones.

## 2. Description of DAS Measurements and Their Transfer Function

DAS measurements are one-dimensional. The direction of the OPD is the axial direction of the optical fiber. For the following expressions, we consider DAS measurements to be in an arbitrary direction. The phase measurements made by DAS systems can be converted to 1D relative displacement $\Delta u_{DAS}$ or average strain ${\hat{\varepsilon }}_{DAS}$ over a gauge length of optical fiber by [3]:

(3) $$\Delta u_{DAS}=\frac{\Lambda \;d\phi }{4\pi n\xi }$$

and

(4) $${\hat{\varepsilon }}_{DAS}=\frac{\Delta u_{DAS}}{g}\;$$

where Λ is the average optical wavelength in a vacuum of the laser used in the DAS system, *n* is the group refractive index of the sensing fiber, ξ is the photoelastic scaling factor for longitudinal strain in an optical fiber (=0.78), and dϕ is the phase change measured by the DAS system. Equations (3) and (4) assume that the strain in the gauge length of optical fiber happens between the origins of scattered light. Scattered light from each end of a gauge length is actually a summation of energy from many scatterers distributed over the light pulse shape. DAS measurements can experience nonlinearity due to localized strain occurring within the distribution of scattering sites from single optical pulse [1,4]. We assume that Equations (3) and (4) are an accurate representation of DAS measurements for the application explored in this paper, though nonlinearity may be a source of noise in the field measurements presented in Section 9.

Since DAS measurements made along an array have dimensions of space and time, its transfer function is also two-dimensional in wavenumber and frequency. We develop the transfer function of DAS, H(f,k), as a multiplication of two transfer functions of independent variables, $H_{f}\left(f\right)$ and $H_{k}\left(k\right)$ such that:

(5) $$H\left(f,k\right)=H_{f}\left(f\right)H_{k}\left(k\right)$$

where *f* and *k* are linear wavenumber and frequency, respectively. H(f,k) transfers the axial strain field along the array, described by its Fourier transform E(f,k) into DAS strain measurements with Fourier transform ${\hat{E}}_{DAS}\left(f,k\right)$ as:

(6) $${\hat{E}}_{DAS}\left(f,k\right)=H\left(f,k\right)E\left(f,k\right)$$

We assume that H(f,k) does not vary in *f* (i.e., a single measurement channel’s response is the same for all temporal frequencies). This is consistent with the assumption made in Equations (3) and (4) that the strain measurements remain linear with optical phase. Therefore, we state:

(7) $$H_{f}\left(f\right)=1$$

The spatial transfer function $H_{k}\left(k\right)$ can be described as a combination of effects from the gauge length, pulse shape, and cable used for the DAS array as:

(8) $$H_{k}\left(k\right)=G\left(k\right)P\left(k\right)C\left(k\right)$$

where G(k), P(k), and C(k) are the transfer functions induced by the application of the gauge length, pulse shape and cable, respectively.

### 2.1. Transfer Function Due to Gauge Length

The application of the gauge length can be represented as convolution of the spatial strain field $\varepsilon_{x}\left(x\right)$ with a scaled rectangular pulse. We use subscript *x* to denote that the strain field is also a function of space and time, though now only variation in space is being described. In the space domain, this is stated as:

(9) $${\hat{\varepsilon }}_{x,g}\left(x\right)=\varepsilon_{x}\left(x\right)*\frac{1}{g}\Pi \left(\frac{x}{g}\right)$$

where ${\hat{\varepsilon }}_{x,g}\left(x\right)$ is the averaged spatial strain field through application of the gauge length, and Π(x) is the rectangular window defined by Bracewell [28] as:

(10) $$\Pi \left(x\right)=\{\begin{array}{cc} 0, & |x|>\frac{1}{2}\\ 1, & |x|<\frac{1}{2} \end{array}$$

This operation is equivalent to multiplication in the wavenumber domain with G(k) defined as:

(11) $$G\left(k\right)=F\left\{\frac{1}{g}\Pi \left(\frac{x}{g}\right)\right\}=\frac{\sin\left(\pi kg\right)}{\pi kg}$$

which has been described previously by Dean et al. [20] as the gauge length effect on DAS measurements made of axially propagating pressure waves.

### 2.2. Transfer Function Due to Pulse Shape

The pulse shape of DAS systems can be approximated as a Gaussian window. Dean et al. [29] presented the filter caused by the pulse shape in the time domain. It is represented here in the space domain for compatibility with the other transfer functions. The pulse shape filter is a Gaussian function in both the space and wavenumber domains. In the space domain, it is:

(12) $$p\left(x\right)=\frac{1}{\sqrt{2\pi }\sigma_{x}}e^{\frac{-x^{2}}{2\sigma_{x}^{2}}}$$

and in the wavenumber domain it is:

(13) $$P\left(k\right)=e^{\frac{-k^{2}}{2\sigma_{k}^{2}}}$$

where $\sigma_{x}$ and $\sigma_{k}$ are the standard deviations in the space and wavenumber domains, respectively, of the Gaussian functions. These are related to the pulse width $h_{p}$ of the DAS system by:

(14) $$\sigma_{x}=\frac{h_{p}}{2.3548}$$

and

(15) $$\sigma_{k}=\frac{1}{2\pi \sigma_{x}}$$

This assumes a pulse width that is defined as the full-width at half maximum (FWHM) of a Gaussian function. Note that most DAS systems define the pulse duration in time $t_{p}$, which can be converted to a spatial length using the speed of light in vacuum c and the group refractive index of the optical fiber through:

(16) $$h_{p}=\frac{t_{p}c}{n}$$

### 2.3. Transfer Function Due to Cable Coupling

The transfer function caused by the cable can be approximated using the strain transfer relationship for layered cables developed by Ansari and Libo [30] and expanded by Li et al. [31]. This solution assumes no slipping at cable material interfaces or within the host media. The assumption requires a cable that is well bonded to both the host media and the optical fiber core. The relationship was originally applied to DAS data by Reinsch et al. [32] for the case when the seismic wavelength and DAS gauge length are equal. However, we suggest a differing approach that incorporates the seismic wavelength and the gauge length separately into the formulation by integrating the governing equation over the gauge length of a DAS system. The ratio of strain experienced by an optical fiber to the uniform axial strain in the cable’s host media can be described as [31]:

(17) $$\zeta \left(x\right)=1-\frac{\cosh\left(\beta x\right)}{\cosh(\beta \frac{L}{2})}$$

where ζ(x) is the ratio of strain transferred as a function of position along the cable, β is the shear lag parameter, and *L* is the length of the bonded fiber section. β is a function of the material properties of a layered cable as:

(18) $$\beta^{2}=\frac{2}{r_{of}^{2}E_{of}\left[\sum_{j=2}^{n}\left(1/G_{j}\right)\ln\left(r_{j}/r_{i-1}\right)+\left(1/G_{1}\right)\ln\left(r_{1}/r_{of}\right)\right]}$$

where $r_{of}$ is the radius of the optical fiber including the core and cladding, $r_{j}$ is the outer radius of layer *j*, $E_{of}$ is the Young’s modulus of the optical fiber, and $G_{j}$ is the shear modulus of layer *j*. We approximate the uniform strain distribution as the strain in one-half of a seismic wavelength, and take the average over a single gauge length of optical fiber. This results in a function of wavenumber as:

(19) $$C\left(k\right)=\frac{1}{g}\int_{-\frac{g}{2}}^{\frac{g}{2}}\left[1-\frac{\cosh\left(\beta x\right)}{\cosh(\beta \frac{1}{k})}dx\right]=1-\frac{2\sinh\left(\beta \frac{g}{2}\right)\mathrm{sech}\left(\frac{\beta }{k}\right)}{g\beta }$$

where C(k) is the ratio of average strain on the optical fiber to average strain on the cable exterior over a gauge length as a function of the linear wavenumber, which can be considered the transfer function.

The full DAS transfer function is then described as:

(20) $$H\left(f,k\right)=\frac{\sin\left(\pi kg\right)}{\pi kg}e^{\frac{-k^{2}}{2\sigma_{k}^{2}}}\left[1-\frac{2\sinh\left(\beta \frac{g}{2}\right)\mathrm{sech}\left(\frac{\beta }{k}\right)}{g\beta }\right]$$

These transfer functions are best understood with a physical example that is given after the presentation of field measurements in Section 9. Next, we develop the relationship between strain measurements made with geophones and DAS because geophones will be used later as a verification tool.

## 3. Relationship between Geophone and DAS Measurements

Geophone measurements are proportional to particle velocity when the fundamental frequency of the geophone’s oscillating proof mass $f_{0}$ is close to the exciting frequency *f* [33]. In this case, the Fourier transform of the velocity experienced by a geophone’s housing $V_{geo}\left(f\right)$ can be described by:

(21) $$V_{geo}\left(f\right)=\frac{V_{mass}\left(f\right)}{J(f)}$$

where $V_{mass}\left(f\right)$ is the Fourier transform of the velocity of the proof mass, which is proportional to electrical potential generated by the geophone and J(f) is the transfer function due to the mechanical and electrical properties of a geophone. J(f) is the transfer function of a damped, forced harmonic motion system with a single degree of freedom defined as [33]:

(22) $$J\left(f\right)=\frac{Sf^{2}}{-f^{2}+2iD\left(f_{0}\right)\left(f\right)+{\left(f_{0}\right)}^{2}}$$

where *S* is a scaling factor to convert from electrical potential to velocity, *D* is the damping of the geophone coil, and *i* is the imaginary unit.

The displacement of the ground can be measured by removing J(f) from $V_{mass}\left(f\right)$ as done in Equation (21) and integrating with respect to time. This is expressed as:

(23) $$U_{f,geo}\left(f\right)=\frac{V_{geo}\left(f\right)}{i2\pi f},\;\;\mathrm{when}\;\;V_{geo}\left(0\right)=0$$

where $U_{f,geo}\left(f\right)$ is the displacement of the ground determined by geophones, expressed in the frequency domain. Now, an array of geophones is considered so subscript *f* is denoted independently from *k*.

The average strain between two geophones is determined by subtracting the displacements measured by a pair of geophones and dividing by their separation distance. This is the same as taking the scaled finite difference of the spatial displacement field as:

(24) $${\hat{\varepsilon }}_{x,geo}\left(x\right)=u_{x,geo}\left(x\right)*\frac{2}{\Delta x}I_{I}\left(\frac{x}{\Delta x}\right)$$

where $u_{x,geo}\left(x\right)$ is the displacement field measured by geophones as a function of space, Δx is the geophone spacing and $I_{I}(x)$ is the odd impulse pair defined by Bracewell [28] as:

(25) $$I_{I}\left(x\right)=\frac{1}{2}\delta \left(x+\frac{1}{2}\right)-\frac{1}{2}\delta \left(x-\frac{1}{2}\right)$$

and δ is the Dirac delta function. In the wavenumber domain, this operation is:

(26) $${\hat{E}}_{k,geo}\left(k\right)=U_{k,geo}\left(k\right)\frac{i2\sin\left(\pi k\Delta x\right)}{\Delta x}$$

Equation (24) is performing the same operation as Equation (9) since:

(27) $$u_{x}\left(x\right)=\int \varepsilon_{x}\left(x\right)dx$$

and

(28) $$\frac{1}{i2\pi k}\frac{i2\sin\left(\pi k\Delta x\right)}{\Delta x}=\frac{\sin\left(\pi kg\right)}{\pi kg},\;\;\Delta x=g$$

The technique of using geophones to take a finite difference of the displacement field has been used in seismic gradiometry for directly inverting wave equations that incorporate spatial gradients of displacement [34,35]. The finite difference process assumes homogeneity over the stencil span (i.e., geophone spacing), and results deviate from the true strain as wavenumbers increase. It is effective when:

(29) $$U_{k}\left(k\right)\frac{i2\sin\left(\pi k\Delta x\right)}{\Delta x}\approx U_{k}\left(k\right)i2\pi k$$

which is a judgement call for a user depending on the accuracy required by the application. For example, Langston [34] suggests a Δx of no larger than 10% of the shortest wavelength to be reliably measured.

In practice, the transformation of the geophone measurements into average strain that are similar to DAS measurements is made by differencing two time-integrated and instrument-corrected geophones spaced by the DAS gauge length as:

(30) $${\hat{\varepsilon }}_{DAS}\left(x,t\right)\approx \frac{\int v_{geo}\left(x+\frac{g}{2},t\right)dt-\int v_{geo}\left(x-\frac{g}{2},t\right)dt}{g}$$

**Figure 1:**
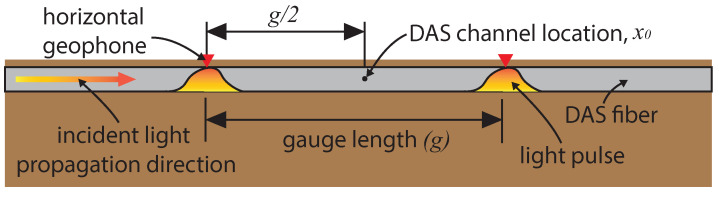
Schematic of DAS sensing as described by the difference between two geophones collocated with the interrogating light pulses at the time of back scattering.

Note that this approximation neglects the impacts of a DAS system’s pulse shape and cable design. Equation (30) is valid only when a DAS channel is centered between two geophones. This is shown schematically in Figure 1. In this case, the scattering centers, or the origin of the Rayleigh backscatter, are at the same location as the first order centered finite difference of the geophones. The two light pulses in Figure 1 show the position of the interrogating light pulses at the time of scattering.

## 4. Directionality of 1D Strain Measurements

The representation of a strain seismic sensor has been presented by Benioff [36]. Lomnitz [37] described the frequency response of this type of system to axially incident plane waves. This was elaborated on by Martin et al. [38] for the case of using strain-rate DAS measurements at the ground surface, neglecting the pulse shape and cable coupling. Martin et al. [38] presents the analytical full waveform representations of pointwise and distributed strain-rate measurements to all types of planar surface and body waves. The expressions found in that work have been time-integrated and simplified by removing the amplitude and oscillatory factors and are presented in Table 1. The purpose of these expressions is to compare the directional sensitivity of different wave types between theoretical point and approximate DAS measurements. The directional sensitivity (*z*) is a multiplier of the solution to the wave equation that determines what strain occurs as a function of angles in the horizontal and vertical planes between an incoming wave and the measurement direction of a sensor, and the wavelength (λ) of the seismic wave. The angles are denoted as θ and α for the horizontal and vertical planes, respectively. This is given for both a point sensor ($z_{\varepsilon }$) and a distributed measurement over a gauge length ($z_{\varepsilon ,g}$). The body waves are also a function of the angle in the vertical plane (α). Note that, for horizontally traveling body waves (i.e., α = 0), the P and Rayleigh expressions are equal, and SH and Love expressions are equal. It is important to note that these expressions assume a far-field, plane wave source, which is not always a valid assumption in active source testing.

If the ratio of distributed strain to pointwise strain is calculated, it can be shown for all surface waves:

(31) $$R_{\varepsilon ,SW}\left(\theta ,\lambda \right)=\;\frac{\lambda sin(\frac{\pi gcos(\theta )}{\lambda })}{\pi gcos(\theta )}$$

and for all body waves:

(32) $$R_{\varepsilon ,BW}\left(\theta ,\alpha ,\lambda \right)=\;\frac{\lambda sin(\frac{\pi gcos(\theta )cos(\alpha )}{\lambda })}{\pi gcos(\theta )cos(\alpha )\;}$$

where $R_{\varepsilon ,SW}$ and $R_{\varepsilon ,BW}$ are the ratios of distributed to pointwise strain for surface waves and body waves, respectively. Notice that:

(33) $$R_{\varepsilon ,SW}\left(\theta ,\lambda \right)=R_{\varepsilon ,BW}\left(\theta ,\alpha ,\lambda \right)=G\left(\frac{1}{\lambda }\right),\;\;\mathrm{when}\{\begin{array}{c} \theta =0^{{}^{\circ}},180^{{}^{\circ}}\\ \mathrm{and}\\ \alpha =0^{{}^{\circ}},180^{{}^{\circ}} \end{array}$$

Equations (31) and (32) can be viewed as extending the gauge length transfer function described in Equation (11) to azimuthal effects of an incoming wave.

**Figure 2:**
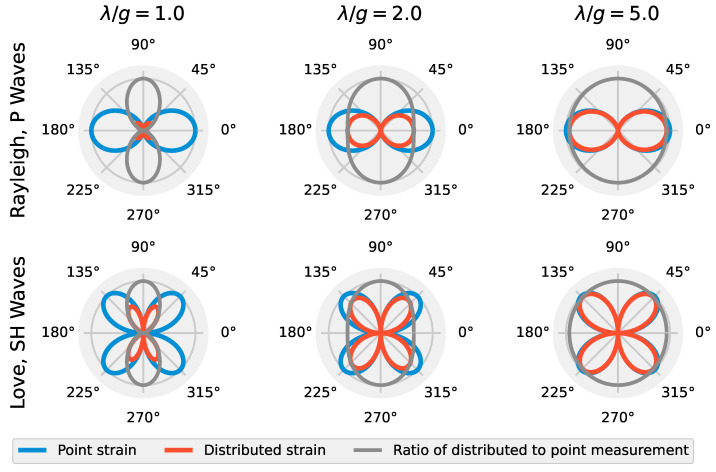
Radial reception patterns for pointwise strain and ideal distributed sensor-measured strain for wavelength to gauge length ratios (λ/g) of 1, 2, and 5 for Rayleigh, P, Love, and SH waves. The patterns are plotted such that the maximum pointwise strain value is 1. In addition, the ratios of theoretical distributed strains and pointwise strains are shown as a function of angle in the horizontal plane (θ), which approaches unity for all wave types and angles as λ/g increases.

It is useful to visualize these expressions as a function of the ratio between signal wavelength (λ) and gauge length of an ideal distributed sensor (i.e., a DAS system that neglects pulse shape and cable design effects). Figure 2 shows this for the case of α = 0° along with the relative amplitude measured between the distributed and pointwise sensors as a function of θ for P, Rayleigh, SH, and Love waves. SV waves have been omitted for brevity. For Rayleigh and P waves at λ/g = 1, the distributed sensor response is zero for incoming waves at 0°, 90°, 180°, and 270°. Love and SH waves always have zeros at 0°, 90°, 180°, and 270°, though the response of the distributed sensor is noticeably distorted from the pointwise strain measurement at low λ/g values. As λ/g increases, the response of the distributed sensor approaches the pointwise strain measurement. This phenomenon is important for using DAS for active source stress wave measurements because surveys need to be designed in such a way to avoid, or to expect, the zeros in the reception patterns for the waves that are being produced when either frequencies are high (i.e., short wavelengths) or when *g* is long. For example, an off-end survey geometry can never be expected to measure Love or direct SH waves. To avoid the zeros in the reception patterns, a variety of source positions need to be used if it is desired to maximize sensitivity to all types of seismic waves, and hence maximize the information gained from a site. In the next section, we develop this idea further with numerical examples of simulated distributed sensor reception with different values of λ/g.

## 5. Numerical Approximation of DAS Measurements

The magnitude response of distributed strain measurements is examined for their dependance on λ/g and directionality through numerically simulated distributed and pointwise strain data. DAS data are approximately simulated by taking the finite difference of the calculated displacement field as would be done for geophone measurements described in Equation (24). This includes evaluating the point displacement at two locations in a model, taking a 1D difference between them in the direction of the virtual DAS array and dividing by their separation distance.

To demonstrate this technique, 3D elastic stress wave propagation was simulated using Seismic Waves, 4th Order (SW4) node-based finite difference code [39]. The model consisted of an elastic half-space solid 200 m-wide, 200 m-long, and 100 m deep. The grid spacing was 0.5 m in all directions, resulting in 32,321,001 total grid points. The material was assigned a shear wave velocity of 250 m/s, compression wave velocity of 433 m/s, and a density of 1800 kg/m${}^{3}$. The velocity values created a material with a Poisson’s ratio of 0.25 (also known as a Poisson solid). There was a free surface top boundary condition and absorbing super grid damping layers surrounding the model to minimize wave reflections at the boundaries. The model was excited using a point force at the top surface with a magnitude of 100 kN defined by a Ricker wavelet. The force was applied with different polarizations, including vertical, parallel to and perpendicular to the 1D strain measurement direction. Each source with horizontal polarization began their Ricker wavelet source signal in the positive *x*- or *y*-direction. The simulations created both surface and body waves with varying wavelengths. Since the model was a Poisson solid, Rayleigh waves propagated at 0.919 times the shear wave velocity, while Love waves propagated at approximately the shear wave velocity [40].

To evaluate the numerical models in terms of λ/g, the fundamental frequency of the source wavelet was set such that the vertical source created Rayleigh waves with a 10 m wavelength (22.975 Hz), and the horizontal sources created Love waves with a 10 m wavelength (25 Hz). Displacement and pointwise strain were evaluated at each grid point at the surface of the model for each run. The displacement values across the surface were then converted to equivalent 1D distributed strain measurements for λ/g of 1 and 5. Essentially, each row of grid points is approximately converted to its own 1D DAS array at the model’s top surface.

**Figure 3:**
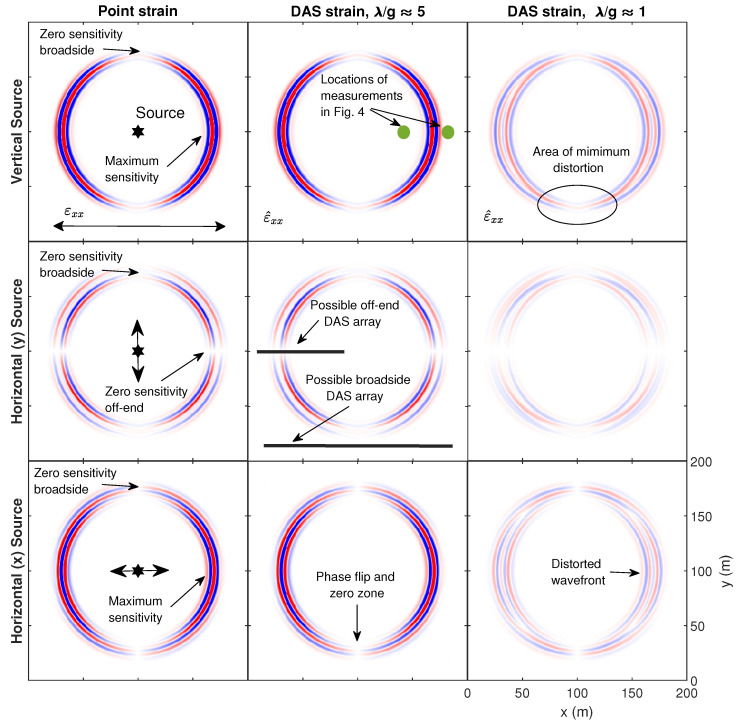
Simulations of normalized pointwise ($\varepsilon_{xx}$) and distributed (${\hat{\varepsilon }}_{xx}$) strain in the *x*-direction at the top surface of a 200 m by 200 m by 100 m elastic half-space model caused by a 100 kN force. The strain is shown as measured by an ideal distributed strain sensing system aligned in the *x*-direction with wavelength-to-gauge length ratios (λ/g) of 5 and 1. This is approximately what DAS would measure neglecting effects from the pulse shape and cable transfer functions. Each source with horizontal polarization began their Ricker wavelet source signal in the positive *x* or *y*-direction. The source is shown centered in the model to demonstrate the theoretical pointwise strain and corresponding distributed measurements at all angles. All tiles are normalized relative to the maximum strain across all simulations. The simulations were conducted using Seismic Waves 4th Order [39].

The pointwise and distributed strain in the *x*-direction as evaluated 0.3 s after source initiation is shown in Figure 3 for the different source polarizations. The simulation results show the normal strain in the *x*-direction, which is denoted in tensor notation as $\varepsilon_{xx}$ for pointwise and ${\hat{\varepsilon }}_{xx}$ for distributed strain. These results can be thought of as approximately what a DAS array oriented parallel to the *x*-axis would measure. The top row displays the vertical source, the center row displays the horizontal source perpendicular to the strain measurement, and the bottom row shows the horizontal source parallel to the strain measurement. The three columns from left to right correspond to pointwise strain, distributed strain with λ/g≈ 5 and distributed strain with λ/g≈ 1, respectively. All amplitudes are normalized relative to maximum strain exhibited across all simulations.

The reception patterns for pointwise strain and distributed strain are evident in the simulation results, including zero sensitivity to perfectly broadside (90° or 270°) sources in the vertical and both horizontal directions. These zero sensitivity zones are labelled in the pointwise strain simulation results. An off-end source-receiver orientation would result in maximum sensitivity to the vertical or *x*-direction horizontal source, which are also labelled, while there would be no sensitivity to the *y*-direction horizontal source. It is important to note the phase-flips that occur for the broadside orientation of the *x*-direction source and the off-end orientation for the *y*-direction source. This observation is similar to the observations made by Luo et al. [41] of broadside microseisms measured by downhole DAS. This results in strain with an opposite sign being measured depending on what side of this phase-flip the receiver is on. This will be evident later in the field-acquired data.

The wavefields are captured well by the approximate DAS data for the case of λ/g≈ 5. The wavefronts have a significantly lower amplitude and become distorted when λ/g≈ 1. This is most evident for the *y*-direction horizontal source. Importantly, the distortion diminishes toward the broadside source-receiver orientation, which is labelled in the top-right tile. This observation is consistent with Equations (31) and (32), and Figure 2, which demonstrates that the ratio of distributed strain to pointwise strain approaches one for angles near θ = 90° and 270° degrees regardless of λ/g.

**Figure 4:**
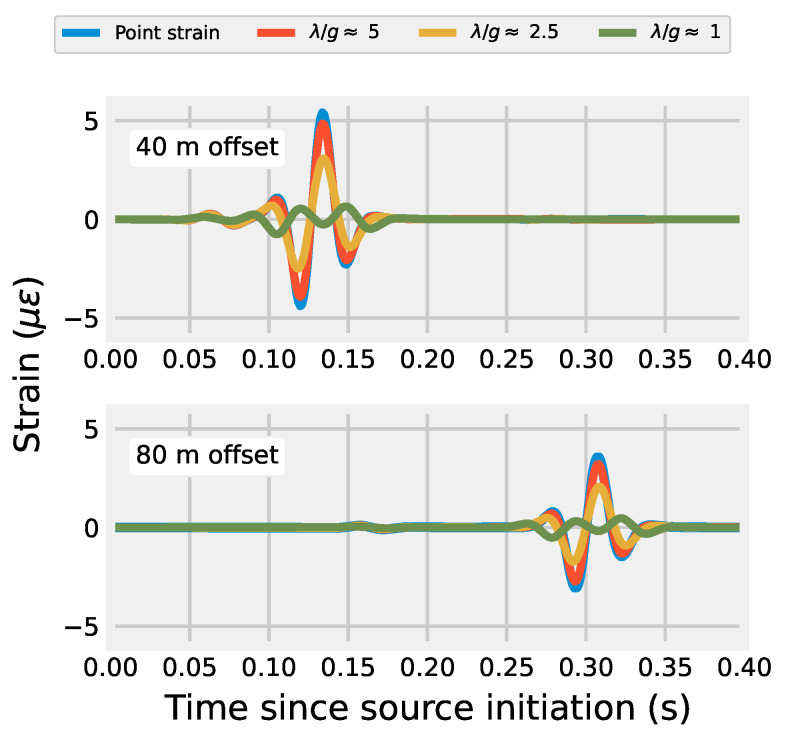
Simulations of ideal distributed strain at the top surface of an elastic model at offsets of 40 and 80 m caused by a 100 kN force in vertical direction. Wavelength-to-gauge length ratios of 1, 2.5, and 5 relative to the Rayleigh wavelength are shown for a virtual DAS array extending radially away from the source position. The simulations were conducted using Seismic Waves 4th Order [39].

The amplitude distortion caused by low λ/g values is shown in Figure 4. Strain simulated by the off-end source-receiver orientation for the vertical source is shown at offsets of 40 and 80 m. The location of these receivers is also indicated in the top-center tile of Figure 3. The λ/g≈ 5 case is very close of the pointwise strain amplitude. The measured amplitude diminishes for the λ/g≈ 2.5 case before becoming completely distorted for λ/g≈ 1. This follows with Equation (11), which describes the gauge length of DAS as a filter in the wavenumber domain. For a monochromatic plane wave solution, the λ/g≈ 1 case would result in zero strain measured (see [24]); the strain is non-zero because the source Ricker wavelet has a non-finite bandwidth. This result is important because it indicates how DAS arrays will fundamentally respond to the most common active source and geometry, the off-end hammer strike. Due to the wide bandwidth of the wavelet generated by an impulse signal like a hammer or weight drop, the ability for DAS to adequately measure the pointwise strain field is a function of λ/g. This can be mitigated if either the user quantifies the amplitude reduction due to the λ/g ratio (i.e., using Equation (11)) for the particular application or, preferably, examines the spatially differenced displacement field as the desired quantity in their forward modelling of a problem. For example, Titov et al. [14] forward-modeled DAS data directly using the spatial difference method for a VSP-type DAS deployment and gained insights for interpreting field data. Since the finite difference is a very simple operation to conduct, it provides a straightforward way to compare DAS and modeled data especially for a deployment where subsurface quantities are known.

## 6. Experimental Campaign

**Figure 5:**
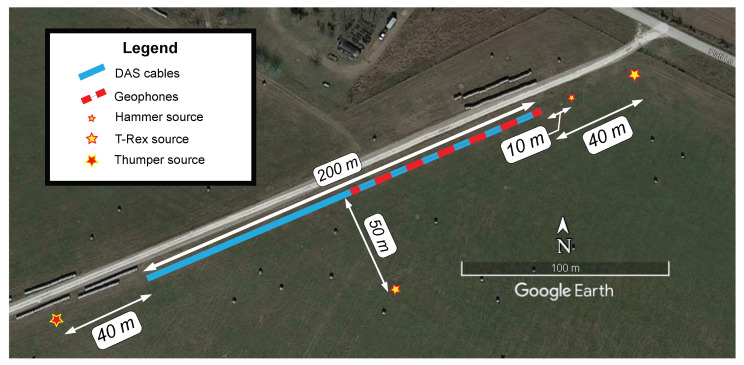
Plan view of the experimental setup at the Hornsby Bend test site along Platt Lane in Austin, TX, USA, where a 94 m geophone array and two 200 m DAS arrays (NanZee and AFL cables) were installed at the same location. The geophones were spaced at 2 m and the gauge length of the DAS system was 2.04 m. The two different DAS cables were spliced together at the far end of the array and interrogated simultaneously. The T-Rex and Thumper vibroseis trucks were used to vibrate the ground 40 m from the arrays in an off-end configuration. T-Rex was also used 50 m from the arrays in a broadside configuration. A sledgehammer was used to strike the ground vertically 10 m from the arrays in an off-end configuration.

An experiment was conducted at the NHERI@UTexas [42] Hornsby Bend test site in Austin, TX, USA to compare active source seismic strain signals recorded simultaneously using DAS and geophones. An overhead view of the test setup is shown in Figure 5. The test used a 94 m-long geophone array (48 geophones at 2 m spacing) directly above two different 200 m-long fiber optic cables buried in a shallow trench. The cables were installed carefully to ensure good coupling with the ground.

**Figure 6:**
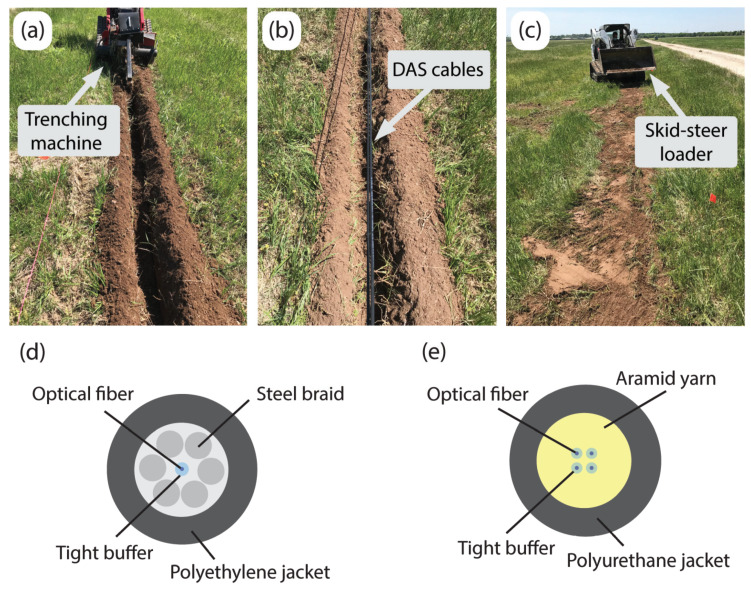
The fiber optic cables were tightly coupled with the ground by: (**a**) using a trenching machine to excavate a trench between 10 and 15 cm deep, (**b**) placing the two cables within the trench next to each other, and (**c**) backfilling and compacting the trench with a skid-steer loader to ensure the soil was densified around the cables. The two installed cables were (**d**) NanZee Sensing Technology’s NZS-DSS-C02 and (**e**) AFL’s X3004955180H-RD.

The cable installation process is shown in Figure 6a–c. First, the end points of the trench were surveyed with a total station and a line was pulled tight to mark the position of the trench. Next, a trenching machine was used to excavate a 10–15 cm deep trench along the marking line (Figure 6a). Once the trench was excavated, the cables were placed side-by-side at the bottom of the trench (Figure 6b). The trench was then backfilled using the same volume of soil that was removed. A skid-steer loader was used to push the soil into the trench and then driven on top to compact the soil (Figure 6c).

Two fiber optic cables cables with tightly buffered fibers were used for DAS arrays. One was NanZee Sensing Technology’s NZS-DSS-C02 (Figure 6d), and the other was AFL’s X3004955180H-RD (Figure 6e). Note that tight buffering only refers to the layer directly outside the optical fiber [43], such that a polymer coating was applied directly to each individual fiber. Outside of that tight buffer, the construction can vary widely from one cable design to another. The NanZee cable has a layer of steel braid that is wound securely around a single tight buffered fiber, which is then encased in a polyethylene jacket. The AFL cable, in contrast, has a layer of aramid yarn surrounding four tight buffered fibers encased in a polyurethane jacket.

The cables were fusion spliced together inside a junction box so that they could be simultaneously interrogated using a single DAS interrogator unit (IU), which for this study was an OptaSense ODH-4. One end of the cables was brought into an instrumentation trailer that housed the IU, geophone data acquisition systems, and vibroseis control electronics. The other end was terminated to reduce end-reflections by fusion splicing on an E2000-style connector and propping open the connector cover so light could escape. The OptaSense ODH-4 IU was configured to have a gauge length of 2.04 m, a channel spacing of 1.02 m, and a pulse repetition rate of 100 kHz for the majority of the acquisition. Gauge lengths of 4.08 and 8.16 m were also used for a few shot records that are used in this study to quantify the value of a smaller gauge length in Section 8. Due to the demodulation scheme used by the ODH-4, which determines the optical phase over four consecutive pulse repetitions, the 100 kHz pulse rate produces a sensing bandwidth of 12.5 kHz. The DAS data were downsampled in real-time to 1 kHz, which involved the application of an anti-aliasing filter and decimating the time series. The downsampling process was handled by the acquisition software provided with the IU. The horizontal geophones used were Geospace Technologies GS-11D with a fundamental frequency of 4.5 Hz. The geophones were housed in PC21 land cases with 7.6 cm aluminum spikes. They were oriented horizontally along the direction of the DAS cables (i.e., in-line) for direct comparison to the DAS data. There were 48 geophones spaced at 2 m for a total array length of 94 m. The geophones were connected to two 24-channel Geometrics Geode seismographs. Geophone measurements were also collected at 1 kHz.

One week after cable installation seismic waves for the study were generated by three different sources: the NHERI@UTexas vibroseis trucks T-Rex and Thumper and a sledgehammer. Wavefields generated by the seismic sources were recorded simultaneously by the DAS and geophone arrays. Figure 5 shows the geometry of the acquisition. T-Rex was used to vibrate the ground vertically as well as in both horizontal directions 40 m from the arrays in an off-end configuration, and 50 m from the arrays in a broadside configuration. T-Rex produced a linear chirp vibration from 3 to 80 Hz lasting 12 s. Thumper was used to vibrate the ground vertically 40 m from the arrays in an off-end configuration but with a linear chirp from 5 to 200 Hz also lasting 12 s. The maximum output forces created by T-Rex and Thumper during their vibrations were 267- and 27 kN, respectively [42]. A sledgehammer was also used to generate seismic signals by striking the ground vertically at 10 m from the arrays in an off-end orientation. Note that this is only a subset of the total data acquisition, which included different source orientations and locations. Other studies including Vantassel et al. [24] have used this dataset, and much of the data are publicly available on DesignSafe-CI [44].

## 7. Experimental Evaluation of DAS Reception Patterns

**Figure 7:**
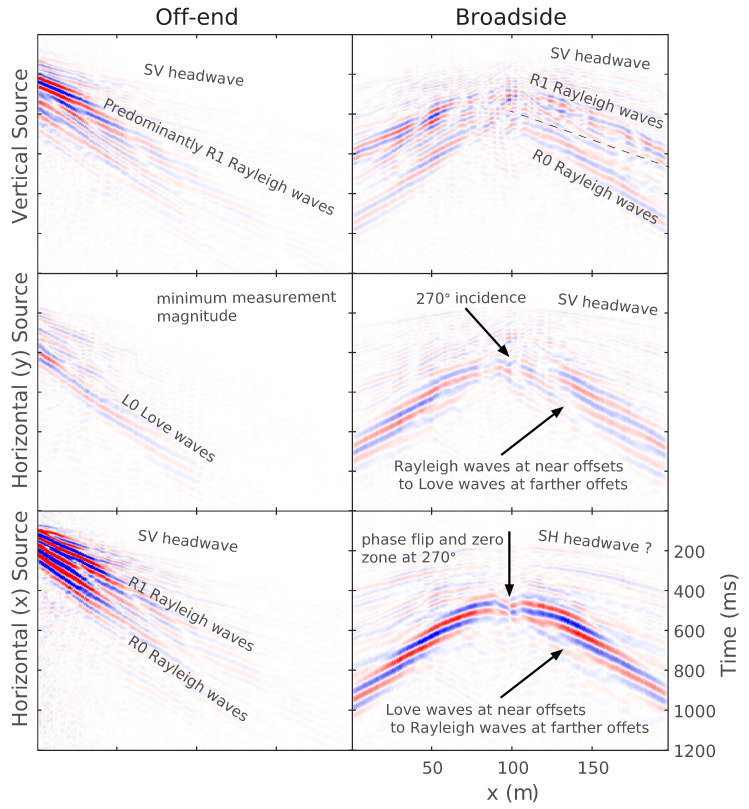
Experimental results using the NanZee cable DAS array of testing the angular reception pattern of DAS subject to active source vibration in both off-end and broadside configuration for vertical and both horizontal directions of excitation using the T-Rex vibroseis source. The DAS array is oriented in the *x*-direction. The off-end geometry is 50 m from the beginning (*x* = 0) of the DAS array, and the broadside configuration is 50 m perpendicular from the midpoint of the 200-m-long DAS array.

First, the DAS-recorded measurements are presented qualitatively to examine the impacts of DAS reception on signal characteristics. As discussed later, both cables were of sufficiently high quality and good coupling such that the cable choice made little difference in signal measurement, so only the NanZee cable is shown in this section. Figure 7 shows seismic recordings from the NanZee cable DAS array using a 2.04 m gauge length during vibrations from T-Rex cross-correlated with the input source sweep signal. This cross-correlation results in a zero-phase source wavelet with center frequency of 41.5 Hz. The data are shown for both off-end and broadside configurations of the vertical and both horizontal directions of excitation using the T-Rex vibroseis source. The DAS array is oriented in the *x*-direction, which is labeled on the horizontal axis. The data are shown normalized relative to the maximum amplitude across all the records for straightforward comparison.

The off-end configurations (the left tiles in Figure 7) give maximum signal amplitude for the vertical source and horizontal source oscillating in-line with the *x*-direction. The horizontal and vertical source orientations create predominantly Rayleigh waves propagating between 250- and 500 m/s with frequencies from 6–30 Hz. An SV headwave is also discernible travelling at approximately 600 m/s with a frequency less than 80 Hz. As expected from the simulations presented in Figure 3, the *y*-direction horizontal source in the off-end orientation produces a much smaller signal received on the DAS array, though there is a slight sensitivity to what is thought to be a fundamental mode Love wave with velocities from 180–350 m/s and frequencies from 8–25 Hz. This sensitivity may be due to imperfect alignment of the horizontal *y*-direction source with respect to the array. As demonstrated in Figure 2, the sensitivity to Love and SH waves picks up abruptly as the angle deviates from 0° or 180°.

For the broadside source position (the right plots in Figure 7), the reception patterns of DAS are more obvious. All three source directions cause a minimum signal reception where the angle of incidence is 270°. However, as shown from the simulations presented earlier, only the horizontal source excited in-line with the *x*-direction sees a phase-flip at the 100 m location. The vertical source induces several modes of Rayleigh waves as well as an SV headwave. The *y*-direction horizontal source creates Rayleigh waves directly perpendicular to the source that transitions to Love waves at farther offsets. The opposite is true for the *x*-direction horizontal source, which creates Love waves perpendicular to the source that transition to Rayleigh waves. There is also a distinct headwave observed in the *y*-direction horizontal source data that may be an SH-type headwave. From visual examination, wavelengths across all vibrations range from approximately 6–80 m indicating that the smallest λ/g value is about 3. However, a more quantitative method for estimating λ/g is proposed next.

## 8. Estimating Wavelength-to-Gauge Length Ratio for Field Data

**Figure 8:**
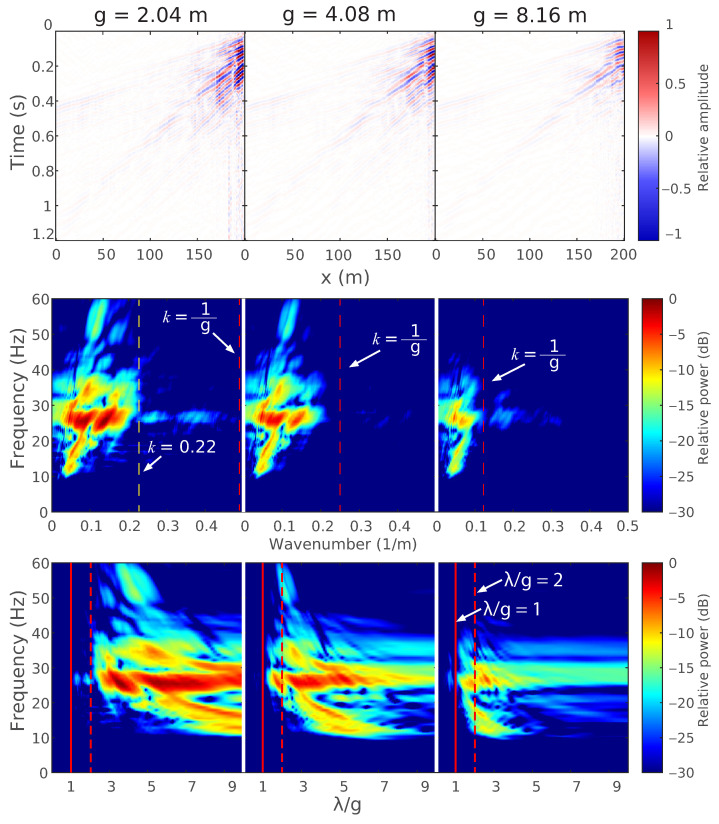
(**Top**) Source-correlated wavefields generated by the Thumper vibroseis truck 40 m from the DAS arrays in the off-end configuration recorded using the NanZee cable and using different gauge lengths of 2.04, 4.08, and 8.16 m; (**Center**) The f−k transforms of the recorded wavefields; (**Bottom**) The remapped f−k transforms as a function of λ/g to identify signal power and estimate the quality of strain measurements.

In this section, vibrations from the Thumber vibroseis truck are used to estimate the λ/g values experienced at the test site for a wide bandwidth (5–200 Hz) signal and various gauge lengths. Figure 8 shows three source-correlated strain wavefield measurements made during 5–200 Hz vibroseis sweeps using gauge lengths of 2.04-, 4.08-, and 8.16 m from left to right. The center row shows the 2D Fourier (f−k) transform of each correlated set of measurements relative to the maximum power in the *g* = 2.04 m case. The bottom row shows *f* as a function of λ/g by remapping the f−k transform.

This type of data visualization allows for the identification of signal power relative to λ/g to assess the data quality for strain quantification. Figure 8 shows that the signal power when *g* = 2.04 m is almost entirely above λ/g = 2, whereas, when *g* = 4.08 m or 8.16 m, the signal power extends lower in λ/g until it abuts the λ/g = 1 line. There is not expected to be much power below λ/g = 1 due to attenuation, but proximity to the λ/g = 1 line acts as an indicator for where the signal energy occurs. Even so, a λ/g = 2 value is not ideal and only results in measuring 63% of the strain amplitude. This can be estimated using Equation (11) for the case of *g* = 2.04 m and λ = 4.08 m as:

(34) $$G\left(\frac{1}{4.08\;m}\right)=\frac{\sin\left(\pi \frac{1}{4.08\;m}\left(2.04\;m\right)\right)}{\pi \frac{1}{4.08\;m}\left(2.04\;m\right)}=0.63$$

However, most of the signal power seems to be above λ/g = 3, which corresponds to 83% of the strain field. Depending on the accuracy desired by a practitioner, acceptable λ/g values may vary.

## 9. Comparison of Strain Measurements from DAS and Geophones

In this section, geophones are used to verify the amplitude of the strain measurements made with DAS. The first processing step of processing the DAS data are to mitigate the laser frequency drift and temperature-induced variation at low frequencies. To do so, a 3 Hz high pass filter was applied to the raw DAS phase measurements and then the measurements were converted to strain using Equations (3) and (4). The geophone measurements were converted from the raw form to particle velocity by compensating for their frequency dependent response as described in Equation (21). Next, the velocity time-series was integrated to achieve units of displacement and the finite difference was applied to the displacement measurements. This is the process described in Equation (30). Then, a DAS channel at about the same location as the center of a differenced geophone pair was selected for comparison.

### 9.1. Uncertainty in DAS Channel Location

**Figure 9:**
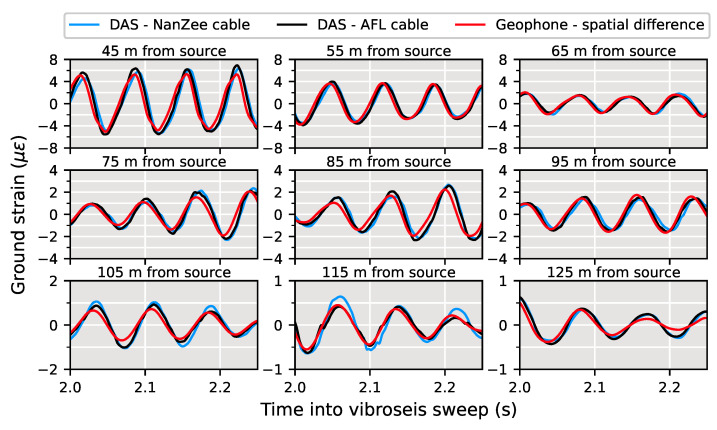
Temporal comparison before shifting of average strain measured with DAS on both the NanZee and AFL cables with geophone strain measurements during in-line shaking by T-Rex 40 m from the array in the off-end configuration performing a 12-s, 3–80 Hz chirp. Each geophone used for strain calculation (differencing) was close to the locations of the scattered optical pulses, but not perfect (see Figure 10). The position indicated is the point between the two differenced geophones (i.e., 75 m indicates the data from subtracting the geophone at 74 m from the one at 76 m).

The result when T-Rex vibrated the ground 40 m from the array in the off-end configuration are shown for six different DAS channels and differenced geophone pairs in Figure 9. For a clear view of the waveforms, 0.25 s of data are shown from 1.50–1.75 s into the 12 s long, 3–80 Hz vibroseis chirp. The amplitudes of both DAS cable measurements match well to the amplitudes of the spatially differenced geophone measurements.

**Figure 10:**
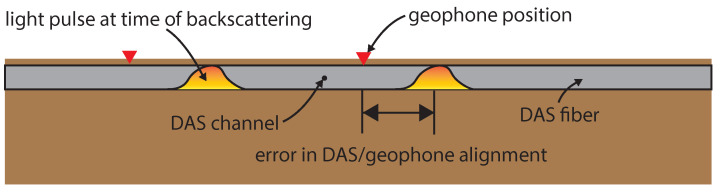
Schematic of the spatial uncertainty of the location of DAS channels in relation to a geophone on the ground surface for a linear DAS array. It is not possible to perfectly locate the scattering positions in DAS arrays so there will be an error between DAS and geophone positioning.

There is a slight phase difference (i.e., time shift) between the time series for all locations, as shown in Figure 9. This is consistent with the phenomena observed by Wang et al. [21] and Egorov et al. [26]. This phase difference is due to the uncertainty associated with the DAS measurement location relative to the geophone positions. As shown in Figure 10, when a DAS system is operated, the sensing fiber is interrogated at a fixed spatiotemporal frequency determined by the digitizer of the DAS system. The measurement channels are evenly spaced, but the exact position is hard to determine. Often a ‘tap-test’ is performed by tapping at a known location and examining what DAS channels respond. However, this is only able to locate the measurement point with a certainty of the gauge length of the DAS system (i.e., +/− one half of a gauge length). Therefore, it is expected that there will be a slight shift in the measurement location between colinear DAS and geophone arrays even when a tap-test is performed.

### 9.2. Correcting Spatial Offset between Geophones and DAS

A method for aligning DAS channels with spatially differenced geophone measurements is proposed here. The method includes spatially up-sampling the DAS data using Fourier method interpolation [45] so that synthetic DAS channels exist every 1 cm along the array. Then, the spatially up-sampled DAS channels are compared to the geophone measurements. This is done by comparing each 1 cm-spaced synthetic DAS channel in the vicinity of a geophone pair with the spatially differenced time-series from that pair. The time series from both DAS and geophones are normalized by their two-norm and then the sum of the square error at each time sample is taken as the misfit $m_{x}$. The minimized value achieves relative alignment between the scattering locations used for DAS and the geophones. The misfit is described by:

(35) $$m_{x}=\Sigma_{j=1}^{n}{\left(\frac{{\hat{\varepsilon }}_{DAS,x}\left[j\right]}{{∥{\hat{\varepsilon }}_{DAS,x}∥}_{2}}-\frac{{\hat{\varepsilon }}_{geo}\left[j\right]}{{∥{\hat{\varepsilon }}_{geo}∥}_{2}}\right)}^{2}$$

where ${\hat{\varepsilon }}_{DAS,x}$ is the time-series of DAS data at relative location x of length n points, ${\hat{\varepsilon }}_{geo}$ is the time-series defined by a spatially differenced geophone pair with a known location, and $m_{x}$ is the misfit calculated for that DAS time-series and geophone pair.

**Figure 11:**
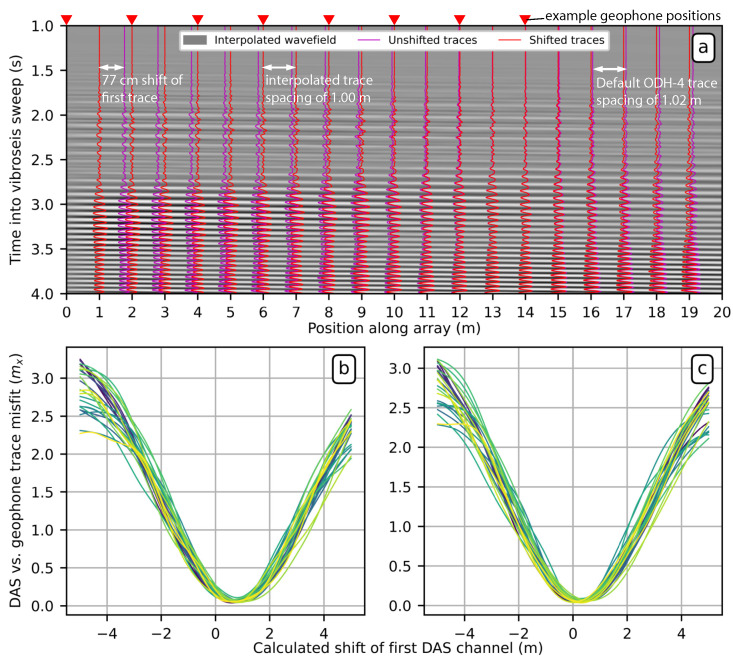
(**a**) Demonstration of the proposed method to align DAS arrays in space with geophones using the NanZee cable array and data from the vertical T-Rex shake 40 m from the array in the off-end configuration. The measured wavefield (magenta traces) is upsampled in space using Fourier interpolation to 1 cm trace separation and shown as the grey variable density background. Each 1 cm-spaced trace is compared to geophone-calculated strain data where the position of the discrete geophones is known. The new 1.00 m spaced set of DAS traces that minimize the misfit between the DAS and geophone measurements are selected (red traces) creating a DAS array where the position of each trace is known. (**b**,**c**) show the minimization result for the relative position of 40 DAS traces relative to geophone pairs for the NanZee and AFL cables, respectively. The green colors are different DAS trace/geophone measurement comparisons shown relative to the shift of the first DAS trace in the arrays. It is necessary to make comparisons relative to the shift of the first trace because the arrays have different spacing (1.02 m vs. 1.00 m), which is ultimately resolved in the interpolation process.

Figure 11a shows an example of how this procedure works using the NanZee cable data from the vertical T-Rex shake 40 m from the array in the off-end configuration. The magenta traces are the first 20 DAS traces spaced at 1.02 m, which is the default trace separation of the ODH-4 IU. The grey variable density image behind the traces is the interpolated wavefield sampled every 1 cm in space. The red traces are shifted to minimize the misfit described by Equation (35) between the DAS and geophone measurements while being constantly spaced at 1.00 m. This process simultaneously corrects for the mismatch in the geophone/DAS channel positions and spacing. Figure 11b,c show the minimization result relative to the position of the first DAS trace in the array for 40 DAS traces and geophone pairs. The results show that the estimated locations of the NanZee cable’s first DAS trace was offset from the geophone positions by about 77 cm, while the AFL cable’s array was offset by about 30 cm. This offset is random, and the AFL cable array just happened to be in better alignment with the geophones. The corresponding new 1.00 m spaced, interpolated DAS channels can be selected to create a shifted DAS array that resolves the phase mismatch with the geophones observed previously (recall Figure 9). It is important to note that the spatial interpolation can only be considered valid for wavelengths larger than twice the trace separation, 2.04 m in this case, based on the Nyquist–Shannon sampling theorem.

### 9.3. Vibroseis Truck Shaking

**Figure 12:**
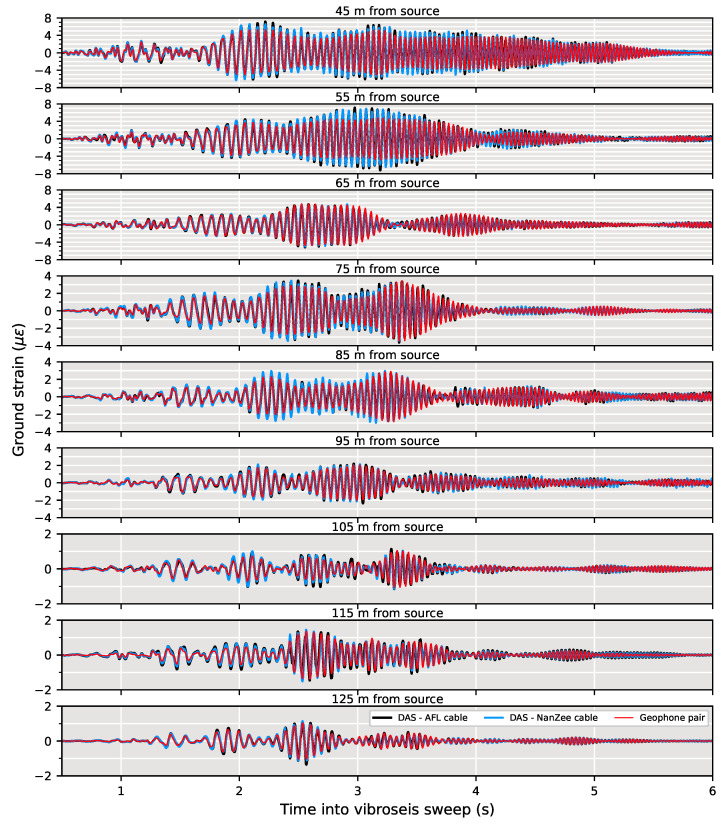
Spatially shifted DAS and geophone data comparisons in units of strain during 0.5–6 s of vertical shaking by the T-Rex shaker truck at a position of 40 m from the array in the off-end configuration performing a 12-s, 3–80 Hz chirp. The DAS data have been spatially shifted from its positions in Figure 9 using the presented Fourier interpolation procedure to match up with the geophone data (77 cm for the NanZee cable and 30 cm for the AFL cable). The distance indicated is the point between the two spatially differenced geophones along the array.

Measurements from the 77 cm shifted NanZee cable and 30 cm shifted AFL cable DAS arrays are compared with the spatially differenced geophones in Figure 12 for a T-Rex vibroseis chirp. The figure shows 0.5–6 s from the time the vibration was triggered at various locations along the array. The amplitude and phase measurements made by DAS are consistent with the measurements made by the geophones. The DAS data measured using both cables tends to have slightly higher amplitudes than the geophone records at near offsets.

**Figure 13:**
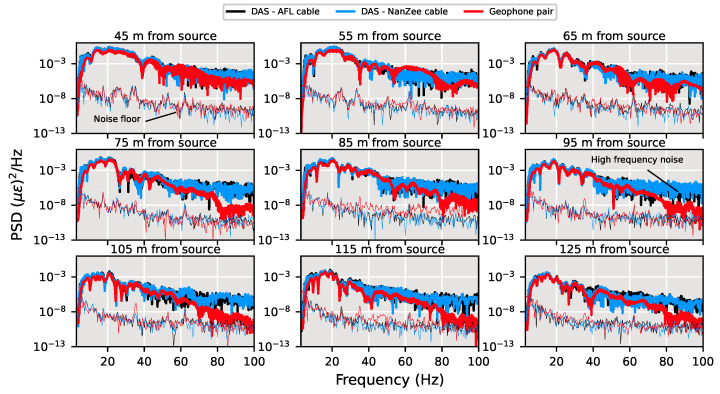
Power spectra of DAS and geophone data comparisons during vertical shaking by the T-Rex shaker truck at a position of 40 m from the array in the off-end configuration performing a 12-s, 3–80 Hz chirp. The noise floor for each DAS cable and the geophones has been calculated from 2 s of quiet time following the vibration.

To gain a more comprehensive picture of the comparison, the time series are visualized by their power spectra. The power spectra shown in Figure 13 are for the DAS and spatially differenced geophones centered at the same locations, as shown in Figure 12 for the frequency range of 3–100 Hz. The noise floor is also displayed for each. The noise floor was determined by taking the power spectrum of the signals during a quiet period when no shaking was happening. The DAS datasets show slightly higher signal power than the geophones across the frequency band, with the DAS showing significantly larger power above 45 Hz. The signal power difference at high frequencies is attributed to phase determination errors and will be explored further later in this section.

### 9.4. Sledgehammer Shot

**Figure 14:**
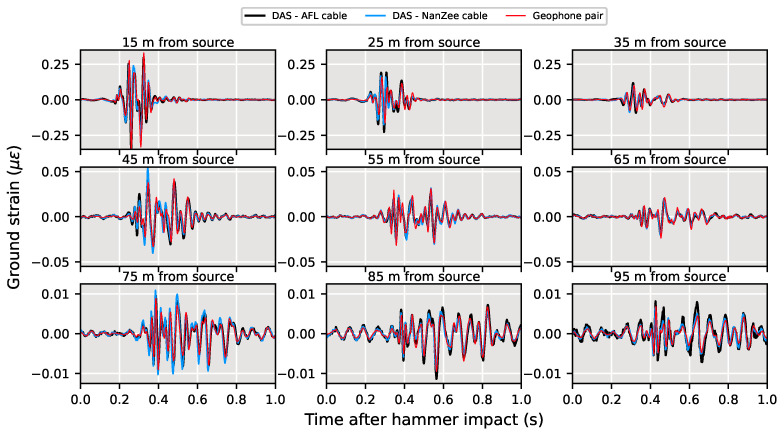
Spatially shifted DAS and geophone data comparisons in units of strain during a vertical sledgehammer strike 10 m from the array. The DAS data have been spatially shifted using the procedure shown in Figure 11 to align with the geophone data. The distance indicated is the point between the two spatially differenced geophones along the array.

The DAS and geophone-measured strain from a vertical sledgehammer striking the ground at 10 m from the arrays is presented in Figure 14. The traces in Figure 14 have been spatially shifted by the same amount as in Figure 12. The measurements are shown for a single sledgehammer strike 0–1 s after impact. The geophone and DAS measurements agree well across the entire array from the hammer strike, indicating that DAS is a viable tool for not only measuring high amplitude vibroseis induced ground shaking but also the less energetic and more traditionally used sledgehammer.

### 9.5. Remarks on Noise in DAS Measurements

Examining the power spectra of the DAS when recording an active source and during quiet time can be a misleading representation of the system’s signal-to-noise ratio. There is an aspect of DAS system noise that is caused by signals that change faster than the phase can be measured, referred to here as phase determination error. This can be thought of as the DAS version of clipping. Even though the DAS system employed a pulse rate of 100 kHz, the rate of strain at each channel can exceed the maximum rate of change the DAS system can capture. This phenomena is described by:

(36) $$d{\dot{\phi }}_{max}=\frac{\pi }{2}\frac{f_{p}}{4}$$

where $d{\dot{\phi }}_{max}$ is the maximum time-rate of change of the optical phase difference that the DAS system can measure and $f_{p}$ is the pulse rate of the ODH-4. Equation (36) is stating that the phase cannot change more than $\frac{\pi }{2}$ radians per effective sample or the phase measurements cannot be correctly unwrapped. The ODH-4 makes a single phase measurement every four pulse repetitions, so this is reflected in Equation (36). This maximum phase rate is equivalent to about 2000 $\frac{\mu \varepsilon }{s}$ for the pulse rate of 100 kHz. Though this seems high, it can be exceeded with high-amplitude shaking.

**Figure 15:**
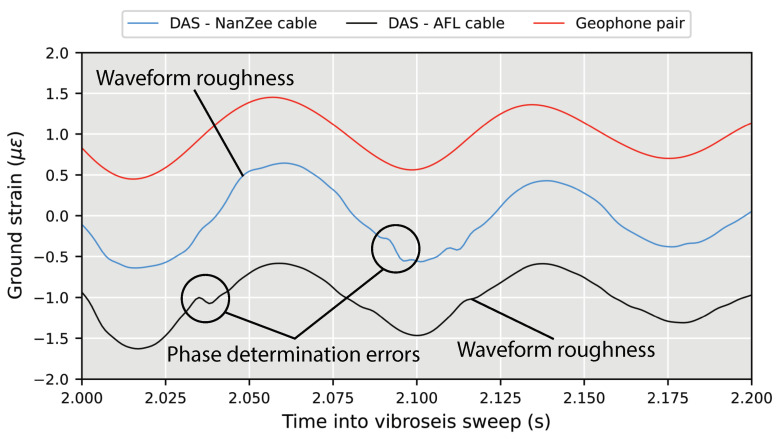
Close-up of the phase determination error problem that causes broadband noise in the DAS measurements at high strain-rates. The example shown is during vertical vibration when the vibroseis was located in the off-end source-receiver geometry 40 m from the beginning of the arrays. The data are shown at 75 m along the arrays (115 m from the source). The time-series have been shifted for examination.

The experimental results indicate that this strain rate was exceeded at times throughout the measurements. Figure 15 shows an example that occurred 2 s into the off-end vertical vibroseis sweep at a location 75 m along the DAS arrays. The DAS data from both cables show abrupt jumps, which may be the result of phase determination error (strain rates higher than the system’s maximum). The geophone data on the other hand is smooth because it does not have this optical limitation.

**Figure 16:**
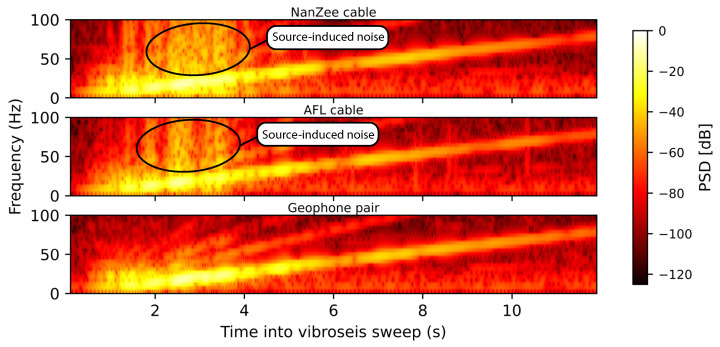
Spectrograms of DAS and spatially differenced geophone time-series 75 m along the arrays (115 m from the source) during the vertical vibration. The spectrogram employs a 206-point FFT over a Hann window with a 205-point overlap between windows. All signals were processed at 1000 Hz sampling rate.

The abrupt jumps manifest as broadband noise in the DAS measurements only when vibration is happening. In addition to the abrupt jumps, there is general waveform roughness in the DAS data. This could be caused by uneven deformation of the soil surrounding the cables, the cables deviating from being perfectly straight or instrument nonlinearity. As mentioned previously, DAS systems behave linearly when the strain only occurs in between the pulse scattering locations. In the case of a 2.04 m gauge length, the scattering centers are very close together and nonlinear behavior may occur. Therefore, the DAS signal power appears to be higher than the geophones at frequencies above about 45 Hz in Figure 14 when it is not actually the coherent signal that has higher power. It is possible to better localize the noise in time by examining spectrograms of the records. Figure 16 shows spectrograms for both DAS cables and the geophone pair at 75 m into the arrays (115 m from the source) during the vertical vibroseis sweep. The noise that extends to high frequencies is concentrated between 2–4 s into the vibroseis sweep, which also corresponds to the time of maximum force output by the T-Rex shaker truck. The spectrogram from the geophone measurements does not show this noise source. The 75 m location selected for Figure 16 is representative of the entire array because this noise persisted at all offsets as indicated by the high frequency power previously shown in Figure 13, though the power of the phase determination error-induced noise diminished with the power of the signal (i.e., remaining relatively constant in SNR).

## 10. Discussion of Signal Amplitude

**Figure 17:**
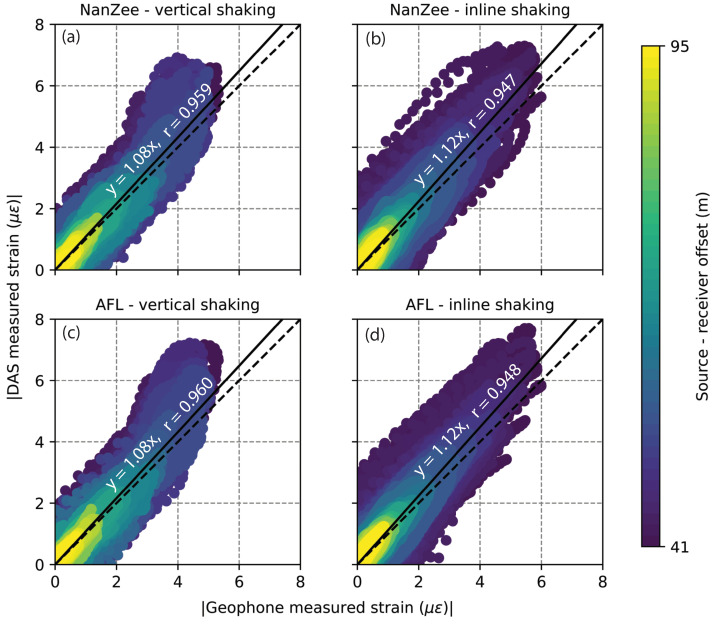
Comparison of strain measurement amplitudes made simultaneously with DAS and geophones over the length of the geophone array during T-Rex shaking in the off-end configuration 40 m from the array for: (**a**) the NanZee cable during vertical shaking; (**b**) the NanZee cable during in-line shaking; (**c**) the AFL cable during vertical shaking; and (**d**) the AFL cable during in-line shaking. The dotted black lines have a slope of 1, and the solid lines are the linear best fit with their equations and correlation coefficients (*r*-values) displayed.

The signals measured with DAS have a statistically larger amplitude than the geophone measurements. Figure 17 shows the comparison of measurement amplitudes made during vibration by the T-Rex source 40 m from the array in the off-end orientation for both cables when the vibration was vertical and horizontal in-line. The vertical shaking resulted in DAS-measured strain values that average 8% higher than the geophone measurements. The horizontal in-line shaking resulted in DAS measurements that average 12% higher than the geophone measurements. The cable choice did not change the results of this amplitude analysis.

**Figure 18:**
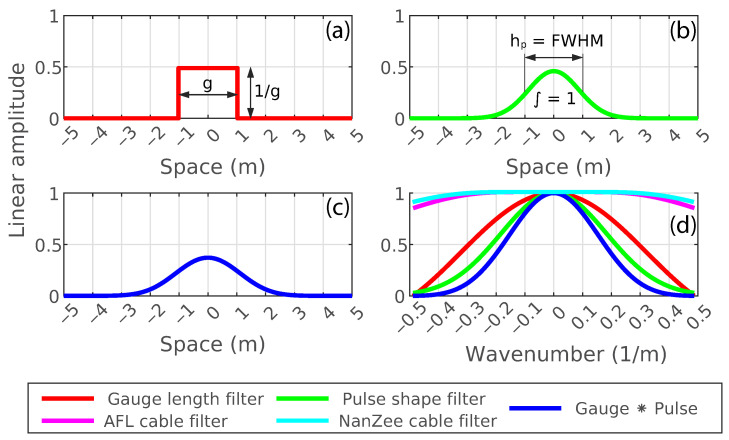
The space domain impulse responses of (**a**) the gauge length filter, (**b**) the pulse shape filter, (**c**) the combined gauge length ∗ pulse shape filters, and (**d**) the transfer functions caused by the gauge length filter, pulse shape filter combined gauge length ∗ pulse shape filters, and the filters for the separate cables.

Using the transfer functions for DAS introduced in Section 2, the response of the field acquisition to spatial waves is shown in Figure 18 as a combination of the gauge length, pulse shape, and cable designs (see Equation (8)). The transfer functions are shown in the wavenumber domain up to the spatial Nyquist frequency determined by the 1.02 m trace separation. The gauge length was 2.04 m, the pulse length $h_{p}$ was 1.02 m (determined by a 10 ns pulse duration time using Equation (16)), and the properties of the two cables are shown in Table 2. Using these properties, the β values as determined by Equation (18) are 1.4 and 1.3 for the NanZee and AFL cables, respectively.

As shown in Figure 18, the gauge length and pulse shape filters dominate the DAS transfer function. The geophone array transfer function for strain measurement is equivalent to the gauge length filter in Figure 18. This approach states that the geophone response should be higher across the entire frequency band than the DAS measurements, but this is not the case. The reason for this slight discrepancy is unknown; however, it is likely from variability in the geophone temporal transfer function described in Equation (22). An advantage of DAS is that the temporal response is flat, requiring no correction. The geophones have a large uncertainty in their correction because it involves damping of a dynamic system *D* and scaling factor *S*. The scaling factor changes with the electrical impedance of the geophones a seismograph. Another source of error in the horizontal geophone measurements is slight vertical coupling. The surface waves that are dominant in the measurements are elliptical in motion, so they have significant vertical energy. Slight orientation of the geophones off of vertical may cause some destructive interference, though this needs to be verified. Considering these sources of uncertainty in the geophone transfer function the data match is quite good. Figure 18 also shows that the cable impact on the DAS transfer functions were minimal, and both the AFL and NanZee cables provide adequate coupling for the spatial frequencies measured. This is confirmed by Figure 17, which shows no measured difference between the amplitudes measured with each cable.

## 11. Conclusions

The potential for using DAS as an engineering tool for applications such as dynamic ground deformation measurement, soil–structure interaction studies, or FWI imaging relies on understanding both the amplitude and phase of the measurements. DAS deployment, quantification, channel positioning, and numerical simulation techniques in this study aim to demonstrate DAS as a viable sensing tool for these types of uses.

The transfer function for DAS was developed encompassing the gauge length, pulse shape, and cable design assuming linear behavior between the fiber optic cable and the ground. A wavelength versus gauge length relationship was developed for assessing strain measurement made with DAS versus the pointwise strain field. This relationship was demonstrated through the transfer function, theoretical reception patterns and by using 3D finite-difference simulations of wave propagation in an elastic solid. It was found that the wavelength versus gauge length relationship is critical for fully capturing pointwise strain waveforms, and this relationship must be understood for effective quantitative deployments where pointwise strain is desired. However, it was demonstrated that strain measured with DAS is easily modeled as a difference of the displacement field over the system’s gauge length, allowing for forward-modeled problems to solve for the DAS measurement directly. In the event that pointwise strain is desired, a method for assessing the wavelength-to-gauge length ratio for field data is demonstrated using a field acquisition and DAS measurements acquired with various gauge lengths.

Experimental comparison of measurements made by geophones and DAS using large vibroseis trucks and sledgehammer strikes was presented. It is shown that DAS measures ground deformation quantitatively with both amplitude and phase that agree with measurements made by geophones. The reception characteristics are consistent with the theory and simulation results including predictable zones of zero reception and changes in the sign (phase-flips) of the measured strain.

This study demonstrates that certain key steps need to be undertaken to ensure the quality of the DAS measurements and their comparison to geophones. Those key steps are: (1) aligning the DAS channels and geophones using a new method presented in this paper, (2) selecting a DAS cable that provides deformation coupling to the internal optical fiber, (3) coupling the cable to the ground through direct burial and compaction, and (4) laser frequency drift mitigation through high pass filtering the DAS measurements. After these steps were taken, the DAS measurements and geophone measurements were shown to be very consistent with each other in both phase and amplitude. Two different suitable cables for direct buried DAS arrays are detailed including a proposed analysis of their transfer functions. A source of broadband noise, phase determination errors, is discussed for its impact on the signal spectra of DAS measurements but is shown to have limited impacts on the waveform comparisons between DAS and geophones.

## Figures and Tables

**Table 1:** Directional and wavelength sensitivity of point and distributed strain measurements to seismic waves (after Martin et al. [38]).

Wave Type	Measurement Type	Quantity	Expression
Rayleigh	point distributed	$z_{\varepsilon }\left(\theta ,\;\lambda \right)$ $z_{\varepsilon ,g}\left(\theta ,\lambda \right)$	$\frac{2\pi }{\lambda }{cos}^{2}\left(\theta \right)$ $\frac{2}{g}cos\left(\theta \right)sin\left(\frac{\pi gcos(\theta )}{\lambda }\right)$
Pressure (P)	point distributed	$z_{\varepsilon }\left(\theta ,\alpha ,\lambda \right)$ $z_{\varepsilon ,g}\left(\theta ,\alpha ,\;\lambda \right)$	$\frac{2\pi }{\lambda }{cos}^{2}\left(\theta \right)\;{cos}^{2}\left(\alpha \right)$ $\frac{2}{g}cos\left(\theta \right)cos\left(\alpha \right)sin\left(\frac{\pi gcos(\theta )cos(\alpha )}{\lambda }\right)$
Love	point distributed	$z_{\varepsilon }\left(\theta ,\;\lambda \right)$ $z_{\varepsilon ,g}\left(\theta ,\lambda \right)$	$\frac{\pi }{\lambda }sin\left(2\theta \right)$ $\frac{2}{g}sin\left(\theta \right)sin\left(\frac{\pi gcos(\theta )}{\lambda }\right)$
Horizontally Polarized Shear (SH)	point distributed	$z_{\varepsilon }\left(\theta ,\alpha ,\lambda \right)$ $z_{\varepsilon ,g}\left(\theta ,\alpha ,\;\lambda \right)$	$\frac{\pi }{\lambda }sin\left(2\theta \right)cos\left(\alpha \right)$ $\frac{2}{g}sin\left(\theta \right)sin\left(\frac{\pi gcos(\theta )cos(\alpha )}{\lambda }\right)$
Vertically Polarized Shear (SV)	point distributed	$z_{\varepsilon }\left(\theta ,\alpha ,\lambda \right)$ $z_{\varepsilon ,g}\left(\theta ,\alpha ,\;\lambda \right)$	$\frac{\pi }{\lambda }{cos}^{2}\left(\theta \right)\;sin\left(2\alpha \right)$ $\frac{1}{g}\frac{cos\left(\theta \right)sin\left(2\alpha \right)}{cos(\alpha )}sin\left(\frac{\pi gcos(\theta )cos(\alpha )}{\lambda }\right)$

**Table 2:** Material characteristics of the cables deployed in the field.

Cable Name	Component	Material	Outer Radius (mm)	Young’s Modulus (GPa)	Poisson’s Ratio	Shear Modulus
NanZee NZS-DSS-C02 *	optical fiber coating buffer reinforcement outer jacket	fused silica acrylate hytrel steel polyethylene	0.0625 0.125 0.45 1.35 2.5	72.4 3.3 1.2 200 1.0	0.17 0.37 0.39 0.28 0.50	31 1.2 0.43 79.3 0.33
AFL X3004955180H-RD ^†^	optical fiber coating buffer reinforcement outer jacket	fused silica acrylate - aramid yarn [46] polyurethane [47]	0.0625 0.125 0.45 2.0 2.75	72.4 3.3 1.2 124 0.50	0.17 0.37 0.39 0.36 0.48	31 1.2 0.43 41 0.17

* Materials and geometry confirmed by cable manufacturer. ^†^ Geometry and material properties estimated by the authors, buffer material name unknown.

## Data Availability

The data presented in this study are openly available in DesignSafe-CI at DOI: 10.17603/ds2-bz52-ep82 (accessed on 4 June 2022), reference number [44].
